# A Rare Case of Emphysematous Pyelonephritis in a Non-diabetic Patient With Staghorn Calculus

**DOI:** 10.7759/cureus.60291

**Published:** 2024-05-14

**Authors:** Sanjay M Khaladkar, Sayali Paidlewar, Sravya Julakanti, Ojasvi Sharma

**Affiliations:** 1 Radiodiagnosis, Dr. D. Y. Patil Medical College, Hospital & Research Centre, Dr. D. Y. Patil Vidyapeeth (Deemed to be University), Pune, IND

**Keywords:** urology, non-diabetic patients, emphysematous pyelonephritis, staghorn calculus, renal parenchyma

## Abstract

Emphysematous pyelonephritis (EPN) represents a severe and acute infection localized in the renal parenchyma and surrounding perirenal area, typically observed in individuals with predisposing factors such as urinary tract obstruction, diabetes mellitus, or compromised immune function. Here, we present a unique case involving a 23-year-old female patient presenting to the emergency department with complaints of discomfort localized to the right side of her abdomen. Despite the absence of diabetes mellitus, the patient was diagnosed with EPN based on clinical presentation and imaging findings. Prompt and effective management was initiated under the care of the urology department, highlighting the importance of early recognition and intervention in mitigating the potential complications associated with this severe infectious process.

## Introduction

Emphysematous pyelonephritis (EPN) is a rapidly progressive and potentially life-threatening condition primarily affecting individuals with type 1 and type 2 diabetes, particularly more prevalent among females [1,2]. This condition is characterized by necrosis of renal parenchyma accompanied by the presence of gas within the urinary system, renal tissue, and adjacent areas [3]. While EPN is most commonly associated with diabetes, there have been rare reports of its occurrence in non-diabetic individuals, particularly those with compromised immune function or obstructive uropathy resulting from factors such as urinary stones, tumors, or sloughed papilla [4].

The term "emphysematous pyelonephritis" was first coined in 1962 by Schultz and Klorfein [5], although the condition was initially described in 1898 by Kelly and MacCallum under the term "pneumaturia" [6]. Since then, our understanding of EPN has evolved, emphasizing its association with diabetes and the potential severity of its clinical course. Early recognition and prompt intervention are crucial in managing EPN effectively, given its propensity for rapid progression and the risk of significant morbidity and mortality. Continued research and clinical vigilance are essential to further elucidate the pathogenesis, risk factors, and optimal management strategies for this challenging condition.

## Case presentation

A 23-year-old female patient presented with a history of persistent right flank pain for one year, along with a background of lithuria and bilateral renal stones. Upon admission, she was observed to be well-oriented and hemodynamically stable, although she exhibited tenderness in the right flank and suprapubic region. The patient denied experiencing fever, hematuria, pyuria, bowel disturbances, weight loss, loss of appetite, trauma, or diabetes mellitus. Laboratory investigations (Table 1) revealed normal results, indicating non-diabetic status. Additionally, the estimated average glucose was 88 mg/dL, and the plasma glucose level was 87 mg/dL. The total leukocyte count was elevated at 12900/µL, with a neutrophil count of 44 and an absolute neutrophil count of 5676 (within the normal range of 2000-7000/µL). HIV testing yielded a non-reactive result. Urinalysis demonstrated the presence of 30-40 pus cells, consistent with a urinary tract infection. Culture sensitivity testing revealed the growth of *Escherichia coli*, confirming the diagnosis.

**Table 1:** Laboratory investigations

Parameter	Observed value	Normal value
Urea	27 mg/dL	17-49 mg/dl
Creatinine	0.56 mg/dL	0.6-1.2 mg/dL
Glycosylated hemoglobin (HbA1C)	4.7%	Less than 5.7%
Average glucose	88 mg/dL	70-100 mg/dL
Total leukocyte count	12900/µL	4,500-11,000/µL
Neutrophil	44%	40-60%
Absolute neutrophil count	5676/µL	2500-6000/µL

**Figure 1:**
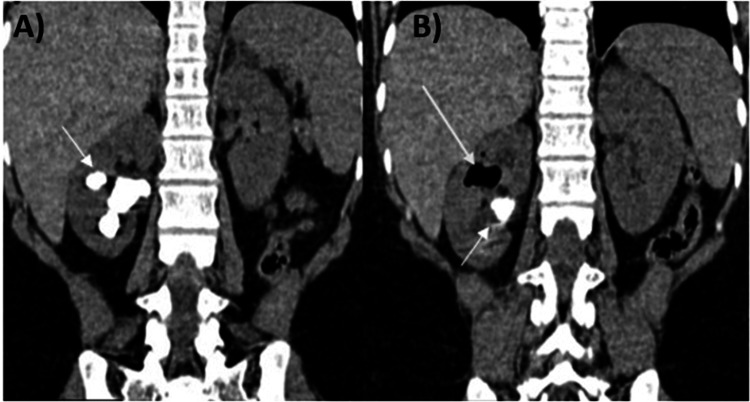
Coronal plane CT images Staghorn calculus in the right renal pelvis (A and B, short arrows). Multiple air foci in the upper and interpolar calyces (B, long arrow).

**Figure 2:**
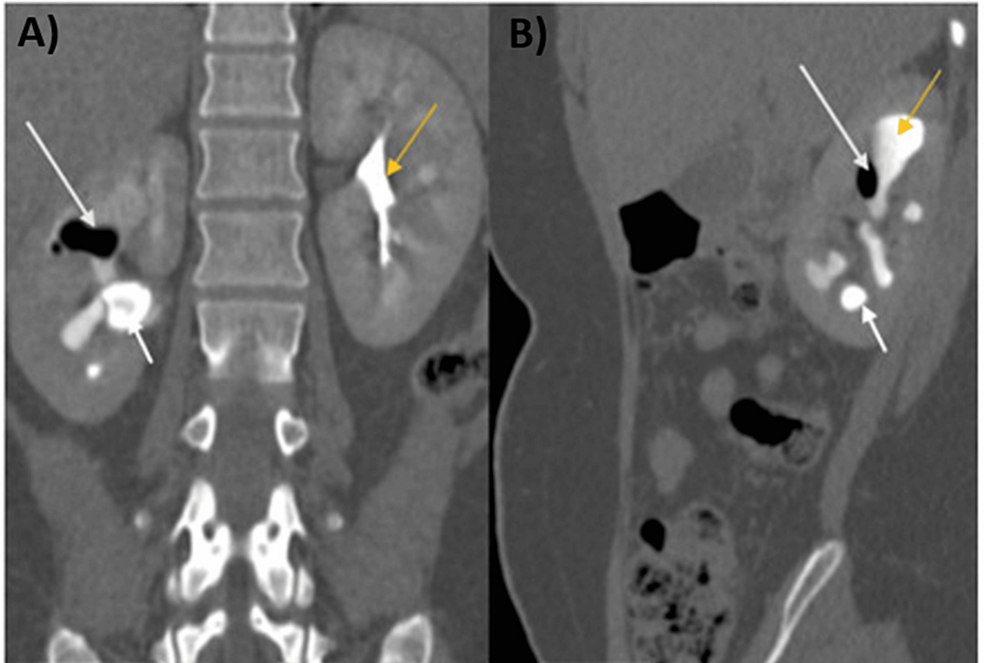
Post-contrast CT images Coronal (A) and sagittal (B) post-contrast CT images show contrast in the pelvicalyceal system (A and B, yellow arrows), multiple air foci in the upper and interpolar calyces (A and B, long arrows), and calculus in the same region (A and B, short arrows).

**Figure 3:**
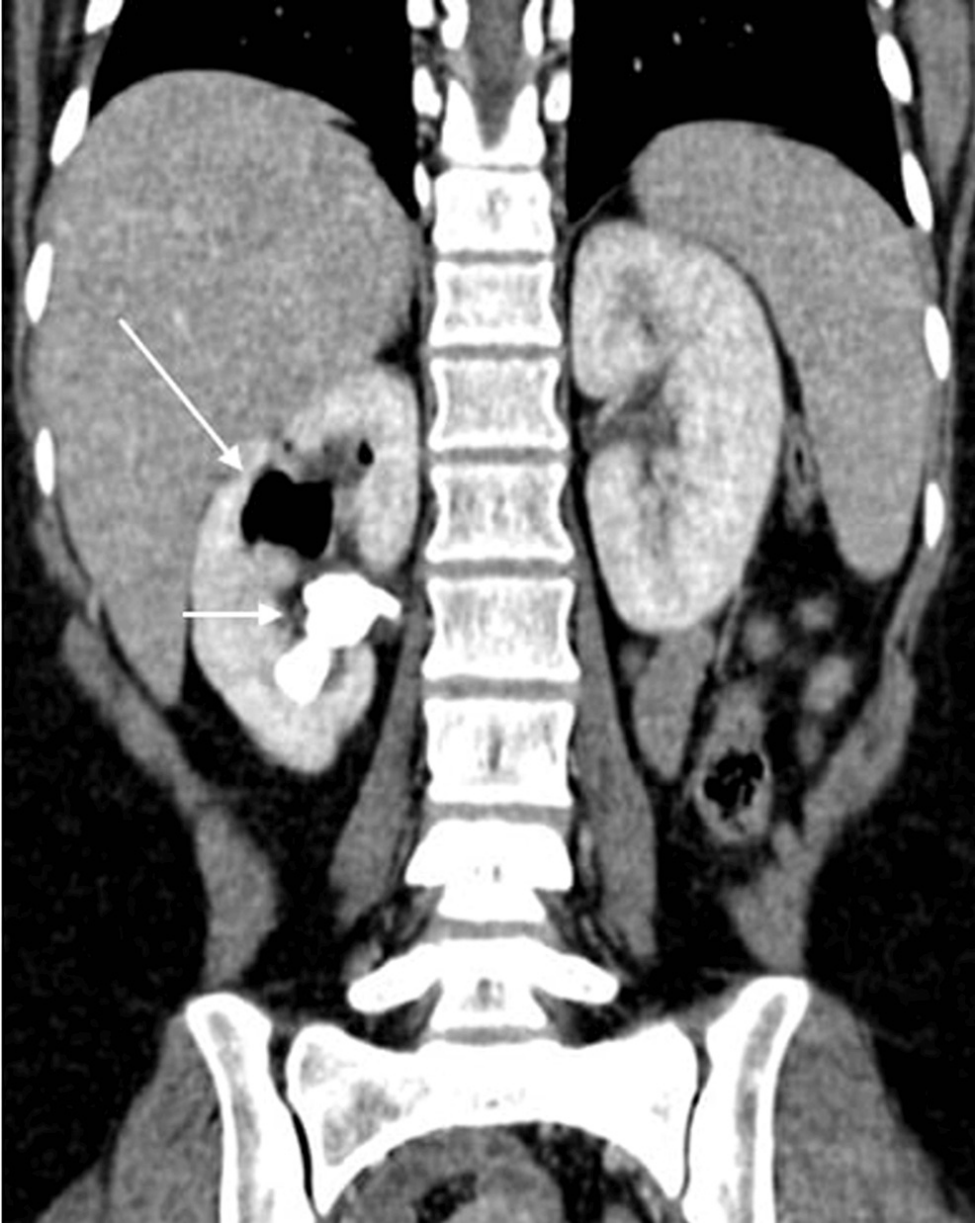
Coronal post-contrast nephogram image Multiple air foci (long arrow) and calculus in the mid-calyx (short arrow).

An ultrasound examination of the abdomen and pelvis conducted outside, prior to presentation at our hospital, had revealed the presence of a large calculus within the right renal pelvis, accompanied by multiple calyceal calculi ranging in size from 5 mm to 10 mm. These calculi caused a mild prominence of the right pelvicalyceal system, while the left kidney appeared normal. Subsequent CT urography further delineated the findings, identifying a staghorn calculus within the right renal pelvis that extended to the mid and lower pole calyces (Figures 1A, 2A) resulting in hydronephrosis (Figures 1B, 2B). The dimensions of the calculus were measured at 28 x 23 mm in the renal pelvis, 14 x 13 mm in the mid-pole, and 15 x 18 mm in the lower pole, with the lower pole calculus exhibiting hyperdensity (CT value of 1200-1300 HU). Additionally, multiple air foci were observed in the upper and interpolar calyces on imaging (Figures 1B, 2, 3).

**Figure 4:**
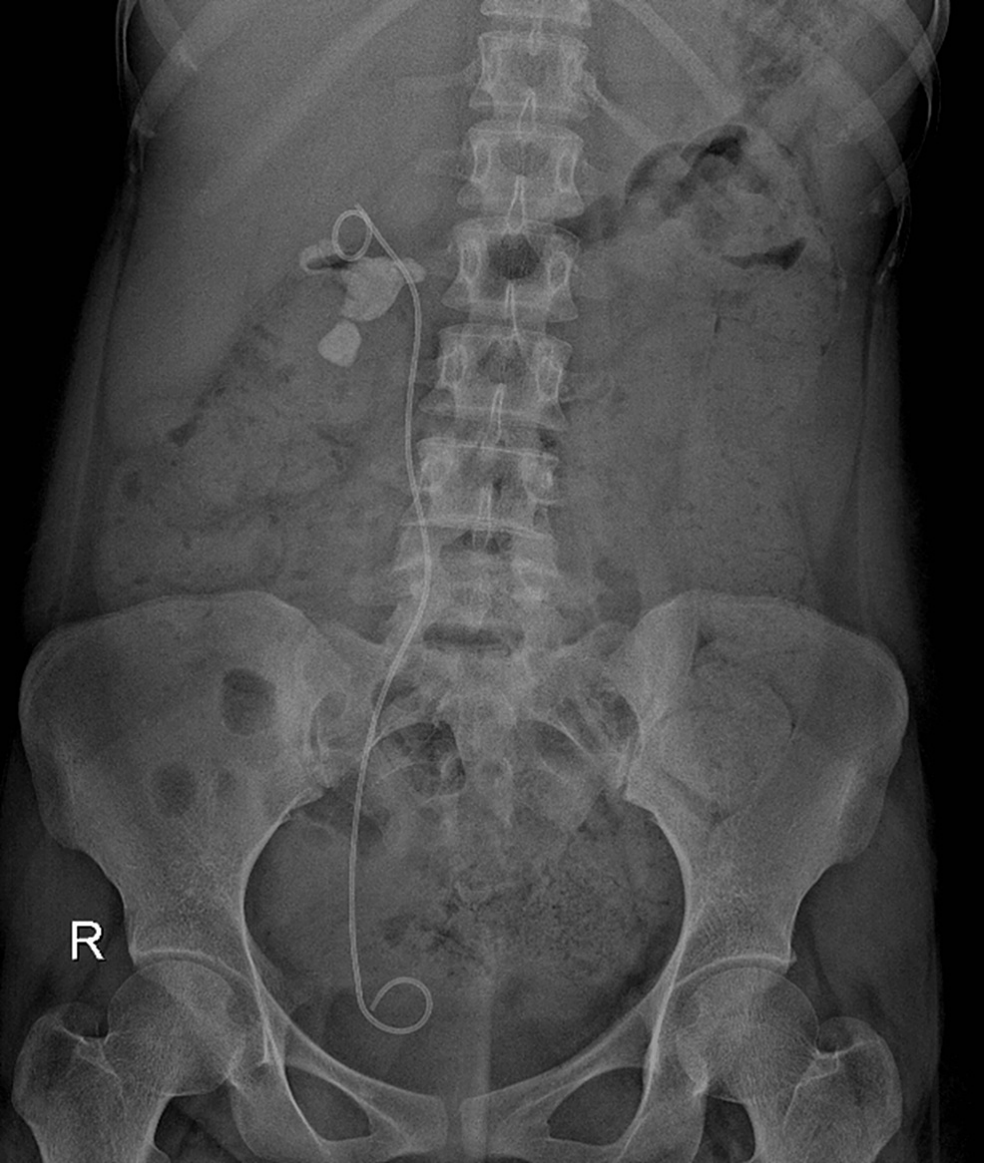
X-ray image after inserting the double J (DJ) stent

**Figure 5:**
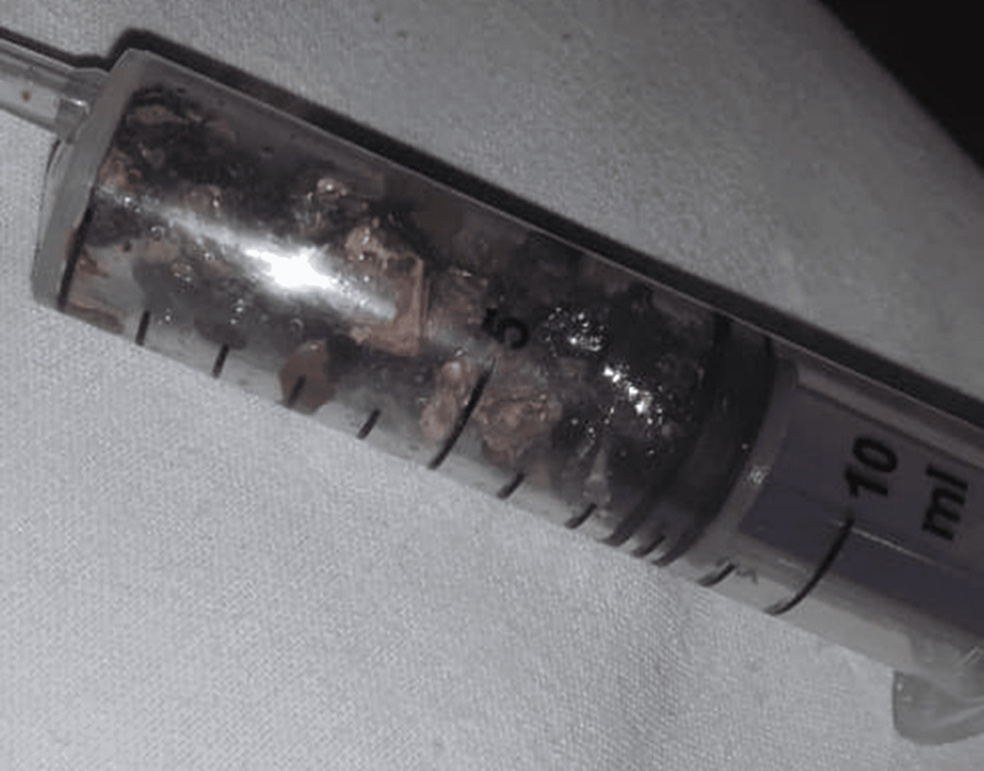
Postoperative image of retrieved fragments of calculi

The patient received a regimen of intravenous medications, including cephalosporin 1 g twice a day, metronidazole 100 cc three times a day, pantoprazole 400 mg once a day, drotaverine, and ondansetron 4 mg three times a day. Additionally, intravenous tramadol was administered in a diluted form with normal saline three times a day for pain management. Amikacin 1 g was administered intravenously once daily for five days. Subsequently, a right double J (DJ) stent placement procedure was performed four days after admission, involving the insertion of a 5/24 DJ stent in a retrograde manner (Figure 4). This was followed by a right percutaneous nephrolithotomy (PCNL) procedure, 19 days after DJ stenting. The staghorn calculus was fragmented with lithoclast. All fragments were removed with stone retrieval forceps. Complete C arm clearance was achieved (Figure 5).

## Discussion

EPN predominantly affects women in their fifth decade, with a ratio of 5.9 to 1 compared to men [7]. This condition is associated with a high mortality rate ranging from 70% to 80% [1,8]. Clinically, EPN presents similarly to acute pyelonephritis, with symptoms including lumbar pain, fever, and vomiting. However, it can rapidly progress to severe complications such as thrombocytopenia, renal failure, sepsis, and shock.

The exact pathophysiology of EPN remains unclear, but it is strongly linked to bacterial infections, primarily caused by gram-negative and anaerobic bacteria such as *Escherichia coli*, *Klebsiella pneumoniae*, *Proteus mirabilis*, and *Pseudomonas aeruginosa* [1]. In patients with diabetes mellitus, uncontrolled blood sugar levels, elevated glycosylated hemoglobin levels, and compromised immune function may contribute to the development and severity of EPN [2]. These factors underscore the importance of managing diabetes and promptly treating urinary tract infections to prevent the onset of EPN.

EPN tends to be more prevalent among diabetic patients, although it can affect individuals across all age groups. This condition, characterized by a rare and potentially life-threatening infection of the kidneys, requires careful diagnosis and management. While medical history, physical examination, and laboratory findings may raise suspicion, the definitive diagnosis of EPN relies on radiological examinations. Patients presenting with diabetes, stone disease, flank pain, and fever are particularly prone to this condition and should undergo thorough evaluation for timely detection and appropriate treatment [4].

Bilateral renal involvement is observed in approximately 5% of cases of EPN [9]. Diagnostic imaging plays a crucial role in confirming the diagnosis, with plain X-rays of the abdomen often revealing speckled gas shadows surrounding the affected kidney. Ultrasonography (USG) can detect blockages and echoreflective gas causing dirty shadowing, although its sensitivity is lower compared to CT in detecting renal gas. In our case, nephrolithiasis was identified as a contributing factor to the condition. Abdominal CT scans are considered the gold standard for diagnosing EPN and assessing renal parenchymal damage.

Huang et al. classified EPN into four categories based on abdominal CT findings. Class 1 is characterized by gas presence only in the collecting system (emphysematous pyelitis), while class 2 indicates gas in the renal parenchyma without extrarenal spread. Class 3A involves gas and/or abscess in the peri-nephric space, while class 3B refers to gas or abscess in the pararenal area. Class 4 includes bilateral EPN or EPN in a solitary kidney. Our case was classified as class 1, with gas confined to the collecting system. The prognosis varies across classes, with classes 1 and 2 typically associated with the most favorable outcomes, including a complete survival rate of 100% and no instances of percutaneous drainage failure. However, mortality rates increase significantly in classes 3A, 3B, and 4, reaching 29%, 19%, and 50%, respectively, underscoring the importance of prompt diagnosis and appropriate management strategies [10].

CT findings play a pivotal role in guiding the therapeutic approach for EPN [11]. When functional kidney tissue is present, the initial treatment typically involves the administration of antibiotics, fluid resuscitation to restore hydration, and management of blood sugar levels, particularly in diabetic patients. Percutaneous renal drainage may also be performed as part of the treatment strategy, either with or without concurrent insertion of a ureteral stent. Studies have shown that percutaneous drainage significantly reduces mortality rates among EPN patients [12]. In cases of severe infection, extensive organ tissue damage, unsuccessful initial therapy, or higher radiological classification on CT scans, nephrectomy may be deemed necessary to mitigate the risk of complications and improve patient outcomes.

## Conclusions

We reported a rare case of EPN in a non-diabetic patient with concurrent staghorn calculus. Typically associated with uncontrolled diabetes and urinary tract blockage, EPN's occurrence in non-diabetic individuals with renal calculi is uncommon. CT stands as the preferred diagnostic modality for early detection and classification of EPN, facilitating prompt intervention and management to mitigate potential complications and improve patient outcomes.
